# Kiwifruit (*Actinidia deliciosa*) changes intestinal microbial profile

**DOI:** 10.3402/mehd.v23i0.18572

**Published:** 2012-06-18

**Authors:** Yuan Kun Lee, Kay Yi Low, Kewin Siah, Lynley M. Drummond, Kok-Ann Gwee

**Affiliations:** 1 Department of Microbiology, Yong Loo Lin School of Medicine, National University of Singapore, Singapore; 2 Department of Medicine, Yong Loo Lin School of Medicine, National University of Singapore, Singapore; 3 Department of Gastroenterology & Hepatology, National University Hospital, Singapore, Singapore; 4 Zespri International Limited, Mount Maunganui, New Zealand

**Keywords:** kiwifruit, polysaccharides, gastrointestinal microflora, faecal lactobacilli, faecal bifidobacteria

## Abstract

**Background:**

Kiwifruit is high in pectic polysaccharides and dietary fiber. This study aimed to find out how the ingestion of kiwifruit will affect intestinal microbiota populations, namely *Lactobacillus*, *Bacteroides*, *Clostridium*, *Bifidobacterium*, and *Enterococcus*.

**Methods:**

Freeze dried kiwifruit (equivalent of two fresh kiwifruits) was given to each of the six subjects daily for four days. Faecal samples were collected before, during and after kiwifruit consumption. The faecal bacteria were enumerated by qPCR and RT qPCR methods.

**Results:**

The effect of the kiwifruit on intestinal microbiota profile varied between individuals; in general, the kiwifruit demonstrated a prebiotic effect of promoting the content of faecal lactobacilli and bifidobacteria (as compared to the baselines of the same individual before consumption) for as long as the fruit was consumed. The effect was however transient, the levels of the two bacteria returned near to that of the baselines upon cessation of consumption.

**Conclusion:**

Kiwifruit is a prebiotic in selectively enhancing the growth of intestinal lactic acid bacteria.

The human intestine has about 10^8^ bacterial cells/ml of luminal content, and the composition of the gut microflora has been found to play important roles in the health and diseases of humans (1–3).

Some slow or non-digestible polysaccharides are termed prebiotics, for they selectively enhance health-promoting gastrointestinal microflora, such as bifidobacteria and lactobacilli, thus confer benefits upon host well-being and health (4, 5).

Some widely consumed fruits and vegetables are high in polysaccharide content. Comparatively, the kiwifruit (*Actinidia deliciosa*) contains high amount of pectic polysaccharides and dietary fiber (6, 7), which has been found to improve immune system and relieve chronic constipation (8, 9). It is possible that the kiwifruit could confer prebiotic effects on intestinal microbiota.

The anaerobic bacteria, namely *Clostridia*, *Bifidobacterium*, and *Bacteroides* (10–12) are dominant intestinal microbiota. Some species of *Bacteroides* were reported to produce metabolites that are carcinogenic and have the potential to aid the development of colon cancer. Other types of undesirable intestinal bacteria such as *Clostridium* may cause pseudomembrane colitis when in active form.

This study focused on five groups of bacteria found in the intestinal flora: *Lactobacillus*, *Bacteroides*, *Bifidobacterium*, *Clostridium*, and *Enterococcus*, and investigated how each of the bacteria group responds to the ingestion of kiwifruit. This would contribute to the understanding of the nutraceutical application of fruits.

## Materials and methods

### Human subjects

It was an open trial involving six healthy Chinese female adults aged 18–25 years. Participants were free from any chronic and recent illness that may compromise the immune system. All participants were kept free from probiotic and prebiotic products such as yogurt and cheese and fermented foods and beverages for 2 weeks before the sampling. Individual informed consents were obtained. The privacy and confidentiality of all data and information collected from trial participants were ensured both during and after the conduct of the trial. Individuals will not be identified in any reports and publications based on the trial data.

### Kiwifruit

The freeze dried kiwifruit was provided by Zespri International Limited, New Zealand.

### The experiment

The six healthy subjects underwent a three-phase experimental regime. Phase 1 is the pre-experiment baseline. In phase 2, the subjects were given 32 g of the freeze dried kiwifruit dissolved in 100 ml water before breakfast. The quantity of freeze dried kiwifruit is equivalent to two fresh kiwifruits. The subjects stopped taking kiwifruit in phase 3 and this represents the washout period. The subjects were refrained from consuming fruits, fruit juices, and yogurts during the experimental period. Each of the three phases consisted of 4 days, whereby fecal samples were obtained daily.

### Faecal samples processing

The stool samples were collected at the household level by each participant. A portion of freshly voided feces was collected into sampling tube and then suspended in RNA*later* (Ambion, USA) to make a 10-fold dilution (v/w) of fecal homogenate. The fecal samples were transported to the laboratory within 12 hours. In the laboratory, the fecal samples were stored at −80°C until use.

Nucleic acids (total DNAs and RNAs) extraction was performed and the intestinal microbiota classified and enumerated by quantitative PCR (qPCR) (11, 12) and reverse transcription-PCR (RT-qPCR) (13, 14).

### Nucleic acids extraction

DNA and RNA were extracted from the fecal samples.

#### DNA extraction

Fecal suspension (200 µl) was vortexed for 3 min with 300 mg glass beads (ca. 0.1 mm) in 300 µl Tris–SDS and 500 µl TE-saturated phenol, to disrupt the bacterial cells. In 400 µl of the supernatant, 400 µl of phenol/choloroform/isoamyl alcohol (25:24:1) was added and shacked vigorously for 45 seconds, after which the supernatant (250 µl) was mixed with 25 µl 3 M sodium acetate (pH 5.2) and 300 µl isopropanol. The mixture was centrifuged at 4°C and the supernatant decanted; 500 µl 70% ethanol was added and mixed, and again the supernatant was discarded. The DNA extract was air dried at room temperature for 30 min and stored at −80°C with 200 µl TE (pH 8.0) until use.

#### RNA extraction

In 200 µl fecal suspension, added 450 µl lysis buffer and 300 mg glass beads (ca. 0.1 mm) and then shacked vigorously for 5 min to disrupt the bacterial cells. To this, 500 µl water-saturated phenol was added and mixed, which was then heated at 60°C for 10 min (hot-phenol method). After which, 100 µl chloroform/isoamyl alcohol (24:1) was added. To 470 µl of the supernatant, 470 µl chloroform/isoamyl alcohol (24:1) was added and mixed. To 400 µl supernatant, 40 µl 3 M sodium acetate (pH 5.2) and 400 µl isopropanol was added and mixed. The supernatant was discarded, the pellet air dried at room temperature for 30 min. To the RNA extract, 0.2 ml nuclease-free water was added and mixed and stored at −80°C until use.

### Bacterial enumeration

Fecal DNA and RNA extracts were subjected to the qPCR and RT qPCR analysis, respectively, using Applied Biosystems PRISM 7500.

qPCR analysis was performed to enumerate the following three predominant bacterial groups: *Clostridium coccoides* group, *Bacteroides fragilis* group, and *Bifidobacterium*. The primers, PCR conditions, and data analysis were according to that of Matsuki et al. (15).

RT-qPCR analysis was performed to enumerate the following two subgroups: *Lactobacillus* and *Enterococcus*. The primers, PCR conditions, and data analysis were according to that of Matsuda et al. (16) and Dubernet et al. (17).

### Statistical analysis

Statistical analyses for significant differences were performed according to parametric, Student *t* text (MPO activity).

## Results and discussion

**Fig. 1:**
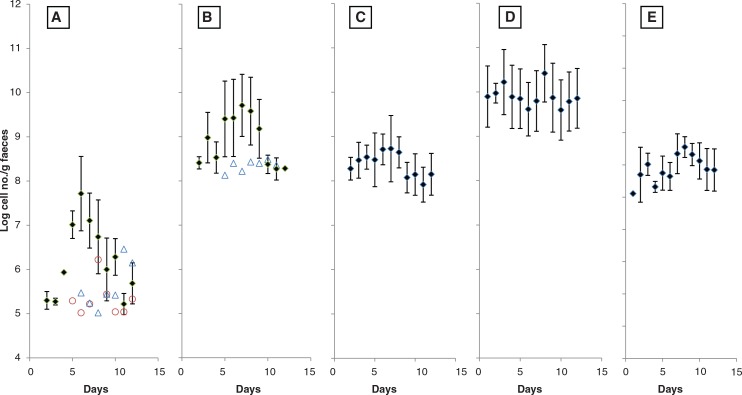
Kiwifruit consumption altered fecal bacteria content. A: *Lactobacilli*; B: *Bifidobacteria*; C: *Enterococci*; D: *Clostridia*; E: *Bacteroides*. Freeze-dried kiwifruit were given on days 4–7. The data points on day 2–4 represent the pre-experiment baseline. The data points from day 8 onward represent the washout period. The vertical bars represent the standard deviations from the average.

Many fruits, seeds, and vegetables contain high polysaccharides and dietary fibers and their potential prebiotic effects have been demonstrated in *in vitro* studies; however, very few human trials have been conducted on these naturally occurring polysaccharides (4, 7, 18, 19). In this study involving six healthy young adults, upon consumption of two kiwifruit equivalent of freeze-dried kiwifruit powder, the fecal content of lactobacilli was found to increase significantly (*p*<0.05) within 24 hours (Fig. 1A), except two subjects who did not respond (plotted independently in open symbols). Interestingly, the lactobacilli content remained at around 10^7^ and 10^8^ cells/g feces despite continuous consumption of the kiwifruit for 4 days. It is not clear if this represents the intestinal ecological niches available to lactobacilli or it is the number of lactobacilli that could be supported by the amount of kiwifruit consumed. The fecal lactobacilli content of healthy Asian reported in previous studies ranged between 10^6^ and 10^8^ cells/g feces (15). If 10^8^ lactobacilli/g feces is indeed the highest level of intestinal lactobacilli attainable, the largest impact for consumption of kiwifruit would be observed in lactobacilli-deficient individuals.

The prebiotic effect of kiwifruit appears to be short term. The content of lactobacilli returned to that of the baseline level rapidly after consumption of kiwifruit was stopped. Continuous consumption of the prebiotic is necessary to maintain a high level of lactobacilli.

A similar pattern of enhancement of intestinal bifidobacteria (Fig. 1B) was also observed. The variation in the bifidobacteria content among the five human subjects (except the non-respondent in open symbol) appeared to be high. Statistical significant different (*p*<0.05) between the experimental and the baseline was only detected after 4 days of kiwifruit consumption (day 8). Intestinal bifidobacteria content is sensitive to prebiotic level in the diet (4). Chinese diet is typically of low meat and high vegetable, and the various vegetables consumed by the subjects daily may contain different oligosaccharide level.

No significant different in the enterococci content was observed before and after consumption of kiwifruit, although its average number seems to be lower during the washout period (Fig. 1C).

For clostridia (Fig. 1D) and bacteroides (Fig. 1E), no significant different (*p*>0.05) was observed between the experimental and the baseline, due to the large standard deviation among the subjects. A general trend of lower cell content for the two bacteria during kiwifruit consumption (days 4–8) is nevertheless apparent. The perturbation in their population could be due to unfavorable environment (organic acid and antibiotic production) created by the lactic acid bacteria (20).

## Conclusion

The study implies that kiwifruit can act as a prebiotic in selectively enhancing the growth of intestinal lactic acid bacteria (lactobacilli and bifidobacteria) and causing perturbation in the population of *Clostridium* and *Bacteriodes*. The extent of their prebiotic effectiveness was depending on individual. The general trend is that kiwifruit consumption enhanced the population of *Bifidobacterium* and *Lactobacillus* within 24 hours, and the effect last only during the consumption of the fruit.
